# Early pregnancy hyperglycaemia as a significant predictor of large for gestational age neonates

**DOI:** 10.1007/s00592-021-01828-1

**Published:** 2022-01-01

**Authors:** Imasha Upulini Jayasinghe, Iresha Sandamali Koralegedara, Suneth Buddhika Agampodi

**Affiliations:** Department of Community Medicine, Faculty of Medicine and Allied Sciences, Rajarata University of Sri Lanka, Saliyapura, 50008 Sri Lanka

**Keywords:** First trimester, Gestational diabetes mellitus, Hyperglycaemia, Large for gestational age, Pregnancy

## Abstract

**Aims:**

We aimed to determine the effect of early pregnancy hyperglycaemia on having a large for gestational age (LGA) neonate.

**Methods:**

A prospective cohort study was conducted among pregnant women in their first trimester. One-step plasma glucose (PG) evaluation procedure was performed to assess gestational diabetes mellitus (GDM) and diabetes mellitus (DM) in pregnancy as defined by the World Health Organization (WHO) criteria with International Association of Diabetes in Pregnancy Study Group (IADPSG) thresholds. The main outcome studied was large for gestational age neonates (LGA).

**Results:**

A total of 2,709 participants were recruited with a mean age of 28 years (SD = 5.4) and a median gestational age (GA) of eight weeks (interquartile range [IQR] = 2). The prevalence of GDM in first trimester (T1) was 15.0% (95% confidence interval [CI] = 13.7–16.4). Previously undiagnosed DM was detected among 2.5% of the participants. Out of 2,285 live births with a median delivery GA of 38 weeks (IQR = 3), 7.0% were LGA neonates. The cumulative incidence of LGA neonates in women with GDM and DM was 11.1 and 15.5 per 100 women, respectively. The relative risk of having an LGA neonate among women with DM and GDM was 2.30 (95% CI = 1.23–4.28) and 1.80 (95% CI = 1.27–2.53), respectively. The attributable risk percentage of a LGA neonate for hyperglycaemia was 15.01%. T1 fasting PG was significantly correlated with both neonatal birth weight and birth weight centile.

**Conclusions:**

The proposed WHO criteria for hyperglycaemia in pregnancy are valid, even in T1, for predicting LGA neonates. The use of IADPSG threshold for Fasting PG, for risk assessment in early pregnancy in high-risk populations is recommended.

**Supplementary Information:**

The online version contains supplementary material available at 10.1007/s00592-021-01828-1.

## Introduction

With physiological changes, the maternal plasma glucose (PG) level behaves differently throughout the trimesters of pregnancy [1]. Hyperglycaemia that is detected during the late second or early third trimester and resolves following delivery is conventionally defined as gestational diabetes mellitus (GDM) [2, 3]. GDM is primarily attributed to increased insulin resistance and increased stress on beta cells during pregnancy [2]. The documented prevalence of GDM varies worldwide for many reasons, including a lack of consensus on GDM diagnostic criteria [4]. The median prevalence of GDM in South Asia is 15% (interquartile range [IQR] = 9.6–18.3). The prevalence of hyperglycaemia in pregnancy (HIP) is 16.9%, and the highest prevalence (25%) has been reported to be in Southeast Asia [5].

There are several issues associated with the diagnosis of HIP worldwide [6, 7]. Primarily, there is a lack of consensus on diagnosis, which leads to delays in detection of and intervention for hyperglycaemia-related pregnancy outcomes [2, 8]. Since O’Sullivan and Mahan’s publication on GDM [9], its definition has changed over time, making it challenging to capture undiagnosed diabetes mellitus (DM) and GDM. Therefore, valid data on worldwide prevalence and outcomes are also scarce [10]. A Hyperglycaemia and pregnancy outcomes (HAPO) study showed a strong relationship between high fasting plasma glucose (FPG) and 1-h and 2-h Oral glucose tolerance test (OGTT) values during weeks 24–32 and adverse maternal-foetal outcomes [10, 11]. Based on the results of the HAPO study, diagnostic criteria for GDM were revised by the International Association of Diabetes in Pregnancy Study Group (IADPSG) [12] and advocated by the World Health Organization (WHO) in their definition for GDM [13][13].

International guidelines recommend that evaluation of DM should be performed early in pregnancy to identify women with pre-existing DM [15]. Screening for DM using IADPSG/WHO criteria before 24 weeks leads to the identification of less severe hyperglycaemic conditions than overt DM. In addition, early lifestyle modification by mothers with hyperglycaemia detected in trimester 1 (T1) has better outcomes [16]. However, a meta-analysis involving over 11,000 mothers reported that interventions for hyperglycaemia in pregnancy were preventive only when applied before the fifteenth gestational week [17]. Nevertheless, most of the studies on GDM are focused on PG fluctuations in the second or third trimester. Recent reviews on GDM highlight the need for prospective studies on the effect of T1 hyperglycaemia on pregnancy outcomes [16, 19].

In pregnancy, hyperglycaemia is associated with many adverse foetal-maternal and neonatal outcomes [18–21], with macrosomia the most common. Foetal macrosomia is known to be associated with many other pregnancy complications [21–24]. Despite this significance, adequate evidence is lacking on the association between T1 FPG and the risk of having large babies at birth, especially from low- and middle-income countries. Against this background, the purpose of the present study was to address the paucity of prospective data with a comprehensive assessment of hyperglycaemia early in pregnancy and its association with pregnancy outcomes, specifically neonate birth weight.

## Methods

We carried out a community-based prospective cohort study, the Rajarata Pregnancy Cohort (RaPCo) [25], in the Anuradhapura district, Sri Lanka. Pregnant mothers who registered at field prenatal clinics were included as study participants. From July to September 2019, 226 RaPCo clinics enrolled over 90% of eligible newly registered pregnant women. Eligibility criteria included pregnant women with singleton pregnancies in T1 (less than 13 gestational weeks) and older than 18 years of age. Gestational age (GA) was determined using ultrasound scan (USS) data, and for those without USS, their last menstrual period (LMP) was used. Exclusion criteria included women with DM (both self-reported or documented evidence), pregnant women on treatment for asthma, psychiatric disorders, autoimmune diseases, cardiovascular events (myocardial infarction and stroke) and those on steroids and hormonal treatments.

A complete clinical interview, anthropometric measures and venepuncture for biochemical assays were performed at baseline. All participants were screened for hyperglycaemia using one-step procedure. Venepuncture was performed by a qualified nursing officer, and 2.5 ml of whole blood was collected in a tube containing sodium fluoride (NaF)/potassium oxalate (K_2_C_2_O_4_) for the FPG test, with universal precautions. An OGTT with a 2-h PG assessment (2.5 ml of whole blood into NaF/ K_2_C_2_O_4_ tube) was conducted with 75 g of glucose dissolved in 300 ml of water. All samples were appropriately labelled and transported in a cool box within 4 h to the Rajarata University Public Health Research Laboratory. Samples were analysed on the same day using an automated Mindray BS-240 Clinical Chemistry Analyzer.

Pregnancy outcome data were collected using three different methods: telephone interviews (TIs), hospital delivery data registers and pregnant mothers’ registers in all public health midwife areas. Data collected through TIs were cross-checked with documented data for accuracy. Several methods were included in follow-up data collection to avoid the problem of similarities in names in the documented data and to capture those who left the area. The main pregnancy outcome variable of concern was neonatal birth weight centile (BWC). For each neonate, birth weight, sex and gestational age at delivery was documented. From these data, the sex-specific BWC was calculated using the INTERGROWTH 21ST standards and tools [26, 27], as this is the most recent tool developed and validated in eight different geographical areas, including South Asia. Large for gestational age (LGA) was defined as a birth weight ≥ ninetieth percentile for a given GA. Small for gestational age (SGA) was defined as a birth weight ≤ tenth percentile for a given GA.

Each woman’s glycaemic status was evaluated using both the WHO [14] criteria for HIP and the conventional DM diagnostic criteria defined by the American Diabetes Association (ADA) [28]. This comparison was done because there is still disagreement about whether to use the conventional criteria for pregnant women in T1, rather than the WHO criteria.

Based on the WHO criteria, GDM was diagnosed if one or more of the following criteria were met: FPG = 92–125 mg/dl and 2-h PG = 153–199 mg/dl following a 75 g OGTT. DM in pregnancy was diagnosed if one or more of the following criteria were met: FPG = 126 mg/dl and 2-h PG = 200 mg/dl following a 75 g OGTT. GDM and DM were collectively labelled as HIP [14]. Based on the ADA criteria (conventional criteria), prediabetes (PD) in T1 was diagnosed if one or more of the following were present: FPG = 100–125 mg/dl and 2-h PG following a 75 g OGTT = 140–199 mg/dl. The diagnostic criteria for DM were similar to the WHO’s criteria for DM in pregnancy.

Prevalence of HIP was reported as a percentage with a 95% confidence interval (CI). Cumulative incidence of outcomes was calculated and stated as per 100 pregnant women in the population. Relative risk was stated with 95% CI for the outcomes studied. In all analytic results, statistical significance was taken as *p* < 0.05. The unconfounded effect of T1 PG levels on LGA was assessed using a binary logistic regression model. Even though the treatment of GDM is a possible confounder, it was not evaluated due to a lack of quality data on treatment of GDM-diagnosed participants.

Ethical clearance for the study was obtained from the Ethics Review Committee of the Faculty of Medicine and Allied Sciences, Rajarata University of Sri Lanka (ERC/2019/07). All the women diagnosed with HIP were given health education and referred to a tertiary care centre for clinical management.

## Results

**Fig. 1:**
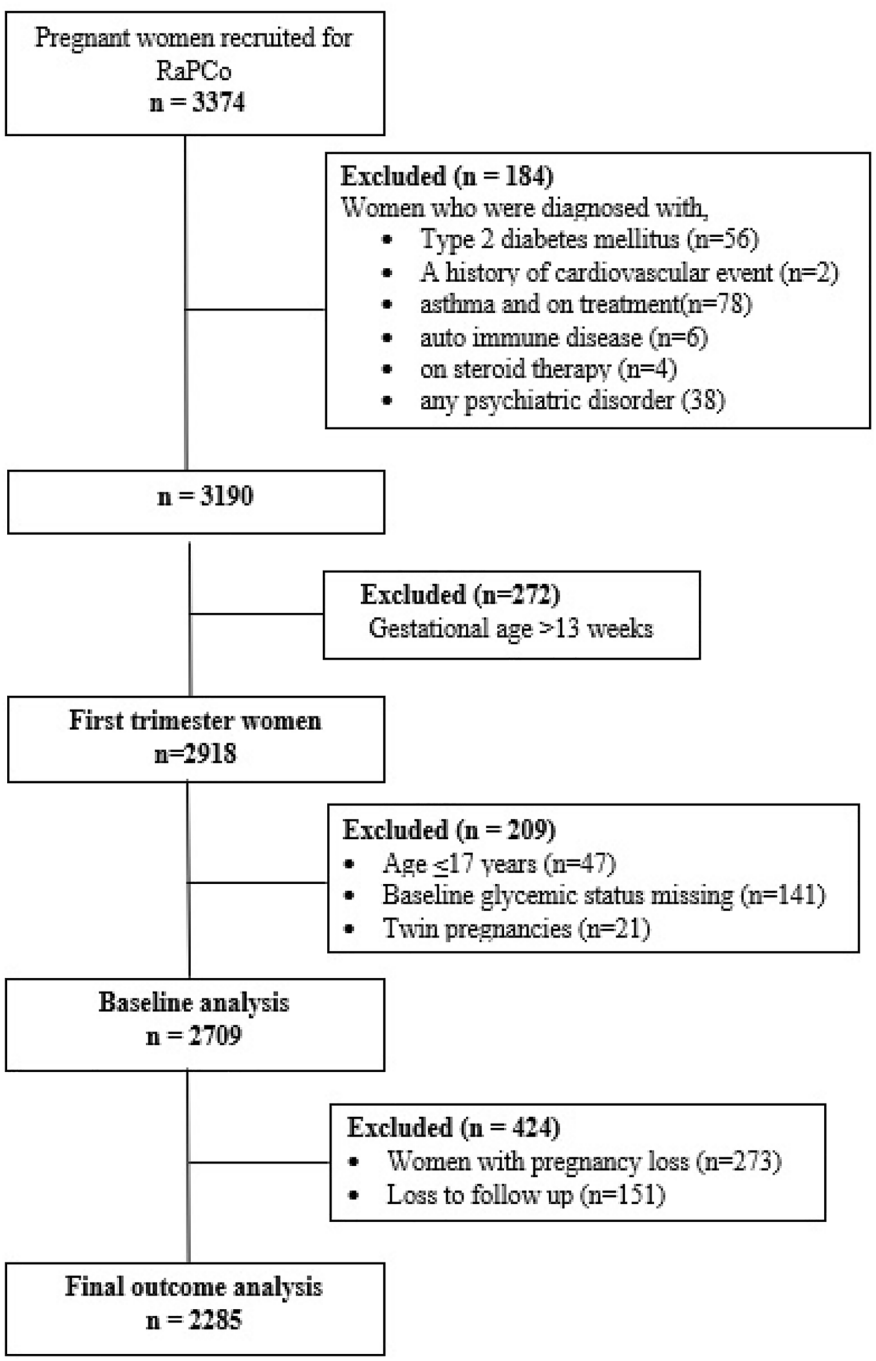
Participant flow of the study

From the original RaPCo cohort, 2709 women were eligible for this study (Fig. 1). Their mean age was 28.0 years (SD = 5.39), and median GA was 8.0 weeks (IQR = 2.0). USS data for GA determination were available in 40.9% of cases, and their median GA was 8.0 weeks (IQR = 3). GA in 59.1% of cases was based on LMP, and their median GA was also 8.0 weeks (IQR = 2).

The mean weight and height of the sample were 55.6 kg (SD = 12.0) and 154.2 cm (SD = 5.7), respectively. The mean waist and hip circumferences were 76.5 cm (SD = 11.5) and 91.7 cm (SD = 10.0), respectively. The mean body mass index (BMI) was 23.4 kg/m^2^ (SD = 4.8).

Table 1 summarises the sociodemographic and anthropometric characteristics of the participants.

**Table 1:** Sociodemographic and anthropometric characteristics of the study cohort (*n* = 2,709)

Characteristic	*n*	%
Ethnicity		
Sinhala	2,353	86.9
Moor	326	12.0
Other	30	1.1
Age at conception (years)		
< 20	151	5.6
20–24	587	21.7
25–29	974	36.0
30–34	651	24.0
35–39	293	10.8
40–44	53	2.0
Gravidity		
1	826	30.5
2	884	32.6
3	673	24.9
4	236	8.7
5 or more	89	3.3
Marital status		
Married	2,686	99.2
Single	23	0.8
Highest level of education		
Up to GCE Ordinary Level	1,597	59.6
Beyond GCE Ordinary Level	1,083	40.4
Gestational age at recruitment (weeks)		
≤ 4	39	1.4
5–6	420	15.5
7–8	1,125	41.5
9–10	788	29.1
11–12	337	12.4
Body mass index		
Underweight	436	16.5
Normal	868	32.9
Pre-obese	429	16.3
Obese class I	647	24.6
Obese class II	255	9.7

n: number of participants, GCE: General Certificate of Education

BMI is classified according to the Asia Pacific Guidelines Underweight: < 18.5 kg/m^2^

Normal: 18.5–22.9 kg/m^2^

Pre-obese: 23–24.9 kg/m^2^

Obese class I: 25–29.9 kg/m^2^

Obese class II: ≥ 30 kg/m^2^

**Fig. 2:**
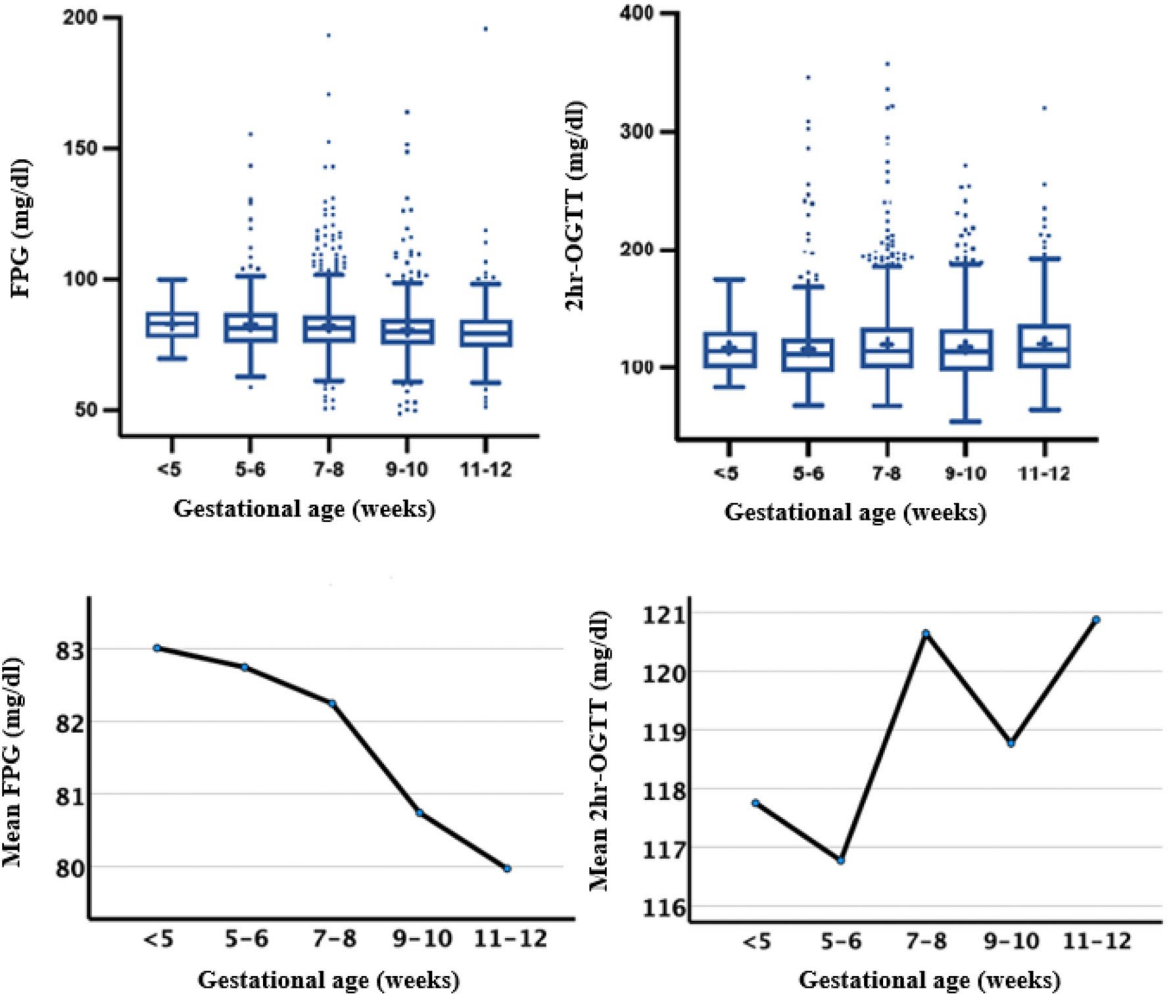
Distribution of fasting plasma glucose (FPG) and oral glucose tolerance test 2-h plasma glucose (2 h-OGTT) values among first trimester pregnant women

The mean FPG and OGTT 2-h PG values among the study participants were 81.6 mg/dl (SD = 11.1) and 119.5 mg/dl (SD = 32.0), respectively. A gradual reduction in mean FPG was observed from 83.0 mg/dl among those with GA < 5 weeks to 80.0 mg/dl among those at 11–12 weeks of GA (Fig. 2). The OGTT 2-h PG values were found to be increasing from week 5 to week 12, but the means fluctuated. Also, a significant negative correlation was seen between GA and FPG in T1 (*r* = − 0.1, *p* = 0.00) but not between GA and OGTT 2-h PG.

The prevalence of hyperglycaemia in T1 of pregnancy using the WHO criteria and conventional criteria was 17.5% (95% CI = 16.1–18.9) and 19.6% (95% CI = 18.2–21.1), respectively (Table 2). The prevalence of GDM using the WHO criteria and of PD using the conventional criteria was 15.0% (*n* = 406, 95%CI = 13.7–16.4) and 17.1% (*n* = 464, 95%CI = 15.8–18.6), respectively. Furthermore, 67 (2.5%) women were identified as having previously undetected DM. Within the study sample, three pregnant women (0.1%) had FPG > 180 mg/dl.

**Table 2:** Glycaemic status of first trimester, previously non-diabetic women with singleton pregnancies according to World Health Organization criteria and American Diabetes Association conventional criteria (*n* = 2,709)

WHO criteria		OGTT 2-h PG, mg/dl				
		Data missing *n* (%)	Normoglycaemia (< 153) *n* (%)	GDM (153–199) *n *(%)	DM (≥ 200) *n* (%)	Total *n* (%)
FPG (mg/dl)	Data missing		18 (0.7%)	–	–	18 (0.7%)
	Normoglycaemia (< 92)	100 (3.7%)	2,118 (78.1%)	195 (7.2%)	20 (0.7%)	2,433 (89.7%)
	GDM (92–125)	21 (0.8%)	143 (5.3%)	47 (1.7%)	26 (1.0%)	237 (8.8%)
	DM (≥ 126)	03 (0.1%)	06 (0.2%)	04 (0.2%)	08 (0.3%)	21 (0.8%)
	Total	124 (4.6%)	2,285 (84.3%)	246 (9.1%)	54 (2.0%)	2,709 (100%)

ADA conventional criteria		OGTT 2-h PG (mg/dl)				
		Data missing *n* (%)	Normoglycaemia (< 153) *n* (%)	GDM (153–199) *n *(%)	DM (≥ 200) *n* (%)	Total *n* (%)
FPG (mg/dl)	Data missing		18 (0.7%)	–	–	18 (0.7%)
	Normoglycaemia (< 100)	114 (4.2%)	2,046 (75.5%)	410 (15.1%)	32 (1.2%)	2,602 (96.0%)
	PD (100–125)	07 (0.3%)	18 (0.7%)	29 (1.0%)	14 (0.5%)	68 (2.5%)
	DM (≥ 126)	03 (0.1%)	03 (0.1%)	07 (0.3%)	08 (0.3%)	21 (0.8%)
	Total	124 (4.6%)	2,085 (77.0%)	446 (16.4%)	54 (2.0%)	2709(100%)

*FPG*, Fasting plasma glucose; *OGTT 2-h PG*, Oral glucose tolerance test 2-h plasma glucose; *GDM*, Gestational diabetes mellitus; *DM*, Diabetes mellitus; *PD*, Prediabetes; *WHO*, World health organization; *ADA*, American diabetes association

All percentage values are rounded up to one decimal place

Among the recruited participants, 273 (10.1%) had pregnancy losses, and 79 (2.9%) were lost to follow-up. Only 2357 (87% of recruited participants) women had live births. Among them, 72 (3.1%) had missing data in at least one variable (birth weight, sex, GA at delivery) required to calculate the BWC. Thus, the final analysis of pregnancy outcome included 2,285 women, for whom the cumulative incidence and relative risks were calculated.

Median GA at delivery was 38 weeks (IQR = 3), and mean neonatal birth weight was 2,940.6 g (SD = 446.9). Among the women who had a live birth, 6.7% (*n* = 159) and 17.0% (*n* = 401) gave birth to LGA and SGA neonates, respectively. Women with DM had a significantly higher risk (Relative Risk (RR) = 2.30, 95% CI = 1.24–4.28) of giving birth to LGA neonates (Table 3). Women with GDM also had a higher risk (RR = 1.84, 95% CI = 1.3–2.5) of having LGA neonates. A significant risk of having SGA neonates was not seen in women with hyperglycaemia according to any criteria in T1. Altogether, using the WHO criteria, women with HIP were two times more likely to have an LGA neonate (RR = 1.98, 95% CI = 1.4–2.7) compared to non-HIP women. In this population, the attributable risk percentage for LGA neonates among women in T1 with HIP was 15.0%.

**Table 3:** Neonatal birth weight centiles in women with hyperglycaemia in first trimester

Glycaemic status in T1		LGA					SGA				
		*n*	Cumulative incidence (per 100)	RR	95% CI		*n*	Cumulative incidence (per 100)	RR	95% CI	
					Lower	Upper				Lower	Upper
WHO criteria	Normoglycaemia (*n* = 1,877)	111	5.9				337	17.95			
	GDM (*n* = 350)	39	11.1	1.80	1.27	2.53	55	15.7	0.88	0.68	1.14
	DM (*n* = 58)	9	15.5	2.30	1.23	4.28	9	15.5	0.88	0.48	1.62
Conventional criteria	Normoglycaemia (*n* = 1,821)	112	6.2				328	18.0			
	PD (*n* = 406)	38	9.4	1.45	1.03	2.06	64	15.8	0.88	0.69	1.12
	DM (*n* = 58)	9	15.5	2.30	1.24	4.28	9	15.5	0.88	0.48	1.62

*GDM*, gestational diabetes mellitus; *DM*, diabetes mellitus; *PD*, prediabetes; *WHO*, World Health Organization; *LGA*, large for gestational age; *SGA*, small for gestational age; *T1*, first trimester; *CI*, confidence interval

Logistic regression modelling was performed to evaluate the unconfounded effect of T1 PG level on LGA. The model included maternal age at conception, ethnicity, gravidity, pre-pregnancy BMI and T1 haemoglobin level. The model was statistically significant, (χ2 [11, *N* = 2,184] = 66.87, *p* < 0.001). However, the model as a whole explained between 3.0% (Cox and Snell R square) and 7.6% (Nagelkerke R squared) of the variance in LGA, and correctly classified 93.0% of cases. Even after the adjustments, T1 GDM (Odds Ratio [OR] = 1.608, 95% CI = 1.071–2.417) and DM (OR = 2.762, 95% CI = 1.274–5.988) were shown to be strong predictors of LGA. In addition, age at conception (OR = 1.064, 95% CI = 1.015–1.095) and BMI (OR = 1.105, 95% CI = 1.069–1.142) also emerged as significant predictors of LGA.

To understand the test-based prediction, FPG and OGTT 2-h PG values were examined separately. Women who were identified as having HIP according to the WHO criteria’s FPG value only (≥ 92 mg/dl) had twice the risk (RR = 2.46, 95% CI = 1.7–3.5) of having LGA neonates compared to non-HIP women. Women with HIP according to the conventional criteria’s FPG value only (≥ 100 mg/dl) had almost thrice the risk (RR = 2.86, 95% CI = 1.7–4.7). A significantly increased risk for having LGA neonates was also found among women who had normal FPG values but elevated OGTT 2-h PG values according to the conventional criteria (RR = 1.53, 95% CI = 1.1–2.2). However, significant risk was not observed in those identified using the WHO criteria (RR = 1.26, 95% CI = 0.8–2.0).

Significant but weakly positive linear correlations were found between T1 FPG and BWC (*r* = 0.1, *p* = 0.00) and neonatal birth weight (*r* = 0.09, *p* = 0.00) (Supplementary Fig. 1). Even though T1 OGTT 2-h PG showed a similar significant correlation with BWC (*r* = 0.08, *p* = 0.00), association with neonatal birth weight was not significant (*r* = 0.03, *p* = 0.17).

**Fig. 3:**
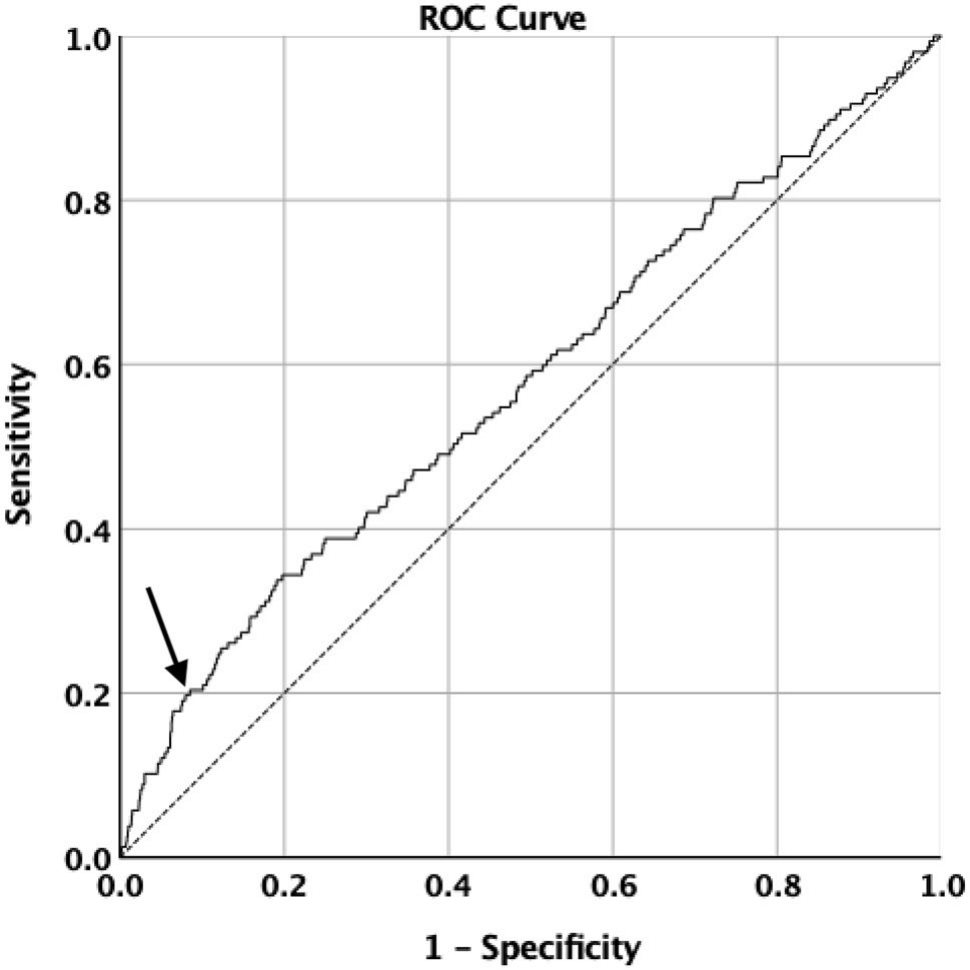
ROC curve for FPG as a predictor of large for gestational age

Since only T1 FPG was found to be associated with BWC, the receiver operator curve (ROC curve) was used to determine the threshold for “high” FPG level in relation to the outcome LGA. The ROC curve (area under the curve (AUC) = 0.58, *p* = 0.001, 95% CI = 0.53–0.63) shows that the FPG threshold is around 92.3 mg/dl, with a sensitivity of 20.4%, a specificity of 91.5% and a sharp change in the curve direction at this threshold (Fig. 3).

## Discussion

This study provides evidence on the occurrence of hyperglycaemia early in gestation, the adverse effect of hyperglycaemia in early pregnancy on birth weight and the validity of the IADPSG FPG threshold for FPG in GDM detection during T1.

It is estimated that over 91.6% of HIP cases occur in low- and middle-income countries. Individual studies have reported the HIP prevalence to vary between 0.4 and 24.3% when using the WHO criteria and IADPSG threshold [29]. However, the studies included in published systematic reviews have been primarily conducted with pregnant women in their second trimester. In studies where T1 PG was assessed, FPG was usually evaluated, not OGTT 2-h PG. Furthermore, the published work on T1 PG assessment was often done retrospectively using routinely available data, which can lead to a selection bias. In the present study, we aimed to address all those methodological issues and showed that the prevalence of T1 HIP is as high as 17.5% (95% CI = 16.1–18.9) using the IADPSG threshold and WHO criteria. This value is significantly higher than the previous estimates of T1 HIP prevalence of 11.4% for Sri Lanka and 11.5% for Asia [30]. Our finding of 19.6% prevalence using the normal adult threshold is similar to the findings of the Sri Lanka Diabetes and Cardiovascular Study [31]. However, that particular study estimates a prevalence of 10.9% for DM, which we have not observed in our study. The reported prevalence of 2.5% of previously undiagnosed DM together with the excluded few pregnant women with DM is less than that reported in previous studies, probably because of the younger age group in our study sample. However, the present study, representing 90% of the target population, provides better estimates for the particular study population.

We noticed a steady decline in the FPG value with increasing gestational age throughout T1, while the OGTT 2-h PG value fluctuated, an observation that was also reported in only a few previously published studies [32]. It is also noteworthy that in our study population, there was a considerable number of women who had normal FPG status but impaired OGTT 2-h PG. Hence, a significant proportion of women with GDM/HIP in T1 was captured only based on their OGTT 2-h PG value (45.5% and 83.2% using the WHO and conventional criteria, respectively). These findings are similar to those of the Early diagnosis of diabetes in pregnancy (EDDIE) study [33]. Even though the EDDIE study researchers performed a one-step procedure at 12–16 weeks of gestation and the cut-off values considered were slightly higher than the IADPSG threshold, the prevalence of early GDM in their population was found to be lower than that in our population (14.9% vs. 17.5%). Nevertheless, 58.5% of all HIP cases were identified using FPG.

The HAPO study showed that there is a significant association between GDM/HIP in the second and third trimesters and having an LGA neonate [10]. Many recent retrospective studies using secondary data or electronic databases in Israel [21], Australia [34], China [11, 35, 36], Spain [16] and other places have shown that this observation is valid for T1 as well. The systematic review and meta-analysis by Farrar et al. [37] showed a graded linear association between glucose concentration and adverse perinatal outcomes, including LGA neonates. This systematic review highlighted that data are missing from LMICs. In our study, we clearly demonstrated that those who were diagnosed with GDM/HIP carried a significantly high risk for having LGA neonates.

Even though the goal of early diagnosis of HIP in pregnancy is to allow better control of glycaemia to reduce the impact of adverse pregnancy outcomes, there are controversies around early identification of GDM/HIP, such as that it has a risk of overtreatment and hence a negative effect on the foetus. The Treatment of booking gestational diabetes mellitus (TOBOGM) pilot randomised control study [38] provided evidence on early diagnosis outcomes: women with treated early GDM had SGA babies (27%) and increased neonatal intensive care unit admissions (36%), while women with untreated GDM had LGA babies (33%). This has stimulated those working in this field to provide more evidence on whether treating early GDM is beneficial or not; hence, directing LMIC health care systems to adhere to more cost-effective and successful screening and treatment protocols for the management of GDM/HIP in pregnancy.

While showing a higher prevalence of HIP in pregnancy, we also noted that the BWC is only associated with the FPG value, not the OGTT 2-h PG value. The FPG threshold for T1 has always been unclear, even after the HAPO study, due to a lack of available evidence for T1. Our study clearly shows that the FPG threshold in T1 (considering the outcome of LGA neonates) is similar to the IADPSG FPG threshold. Hence, the WHO criteria for GDM, irrespective of the trimester, is more valid, at least in Asian settings. To our knowledge, the threshold for T1 has not been previously evaluated in the medical literature using a prospective design.

Though these associations were demonstrated using multivariable analysis, one important variable is missing from our analysis. We referred all women with HIP for specialist care, yet the details of their management were not available. Previous studies have shown that treatment is a significant predictor; thus, this missing variable should be considered in interpreting these data. Another main limitation of this study is that we used FPG and OGTT 2-h PG in the study, omitting the OGTT 1-h PG value. This may lead to an underestimation of the prevalence of HIP but will not affect the associations observed.

In conclusion, we found that there is a significant prevalence of GDM/HIP in T1 among this South Asian population, and that they have a significantly increased risk of giving birth to LGA neonates. We further demonstrated that the WHO criteria with the IADPSG threshold, irrespective of trimester, are valid for predicting LGA. Although more information is required before deciding to standardise the performance of the OGTT in T1 or not, using the IADPSG FPG threshold (92 mg/dl) for risk assessment early in pregnancy is strongly recommended. Balancing the cost effectiveness and clinical importance of T1 glycaemic evaluation also requires the generation of more evidence on other pregnancy outcomes.

## Supplementary Information

Below is the link to the electronic supplementary material.

Supplementary file1 (PDF 80 KB)

## Data Availability

The data that support the findings of this study are available from the corresponding author upon reasonable request.
