# Supplementary material for: Early pregnancy hyperglycaemia as a significant predictor of large for gestational age neonates
Source: Acta Diabetol. 2022 Jan 1;59(4):535–43. doi: 10.1007/s00592-021-01828-1 (PMC8917036; doi:10.1007/s00592-021-01828-1)
Supplement: Supplementary file 1 — Supplementary file1 (PDF 80 KB) [file 592_2021_1828_MOESM1_ESM.pdf]

Early pregnancy hyperglycaemia as a significant predictor of large for gestational age neonates

Journal: Acta Diabetologica

Authors

Imasha Upulini Jayasinghe[1], Iresha Sandamali Koralegedara[1], Suneth Buddhika Agampodi[1]

Affiliations

[1] Department of Community Medicine, Faculty of Medicine and Allied Sciences, Rajarata University of Sri Lanka, Saliyapura, Sri Lanka, 50008

Corresponding author:

Imasha Upulini Jayasinghe

hmiujayasinghe@gmail.com

T +94 252 226 252 | M +94 714 863 374

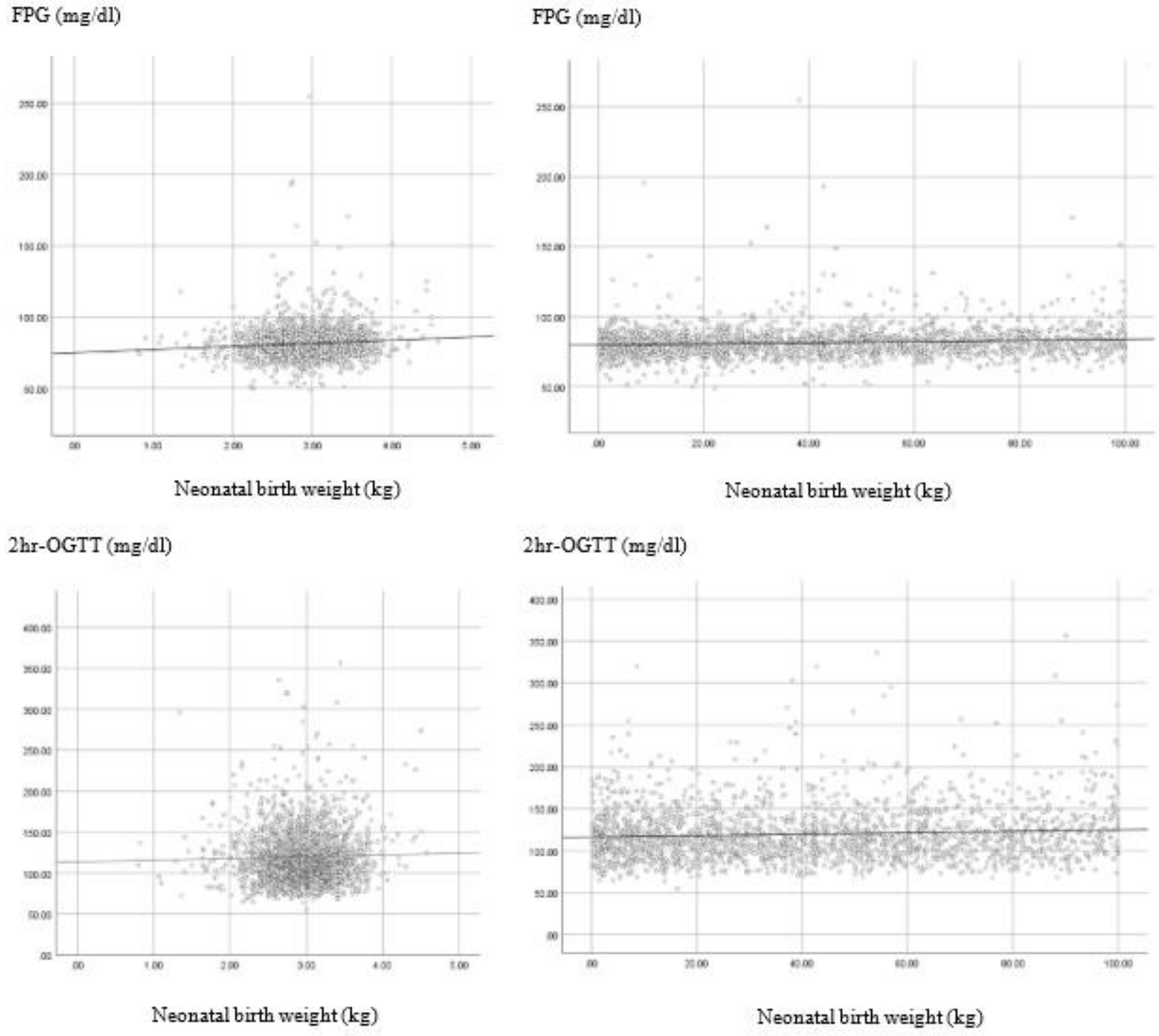

Supplementary Fig. 1 Correlation of neonatal birth weight and weight centile with first trimester fasting plasma glucose (FPG) and oral glucose tolerance test 2-h plasma glucose (OGTT) value
